# Individual corporate reputation and perception of collective corporate reputation regarding stock market investments

**DOI:** 10.1371/journal.pone.0257323

**Published:** 2021-09-14

**Authors:** Anna Blajer-Gołębiewska

**Affiliations:** Faculty of Economics, University of Gdansk, Sopot, Poland; University of Almeria, SPAIN

## Abstract

There are two different concepts of corporate reputation grounded in individual and collective perceptions, respectively. The aim of this study was to identify how these two ways of conceiving of corporate reputation affect investors’ decisions about whether or not to buy stock in a given company. As this problem tackles individual decision-making processes, we designed and applied an incentivised economic experiment based on vignette studies and focused on individual decisions of single investors. Subjects took part in an online game that imitates stock exchange conditions and that concerns corporate reputation and investing. We found that the individual propensity to invest is not directly based on an investor’s perception (rooted in historical share price and other objective metrics) of a firm’s reputation but rather on an investor’s subjective recognition of collective corporate reputation in the market. This suggests a need to rethink the popular measures of corporate reputation in the context of studies of stock market investor decisions.

## Introduction

There is a growing interest in corporate reputation in light of the role that positive corporate reputation plays in enhancing cooperation with clients and banks [1–6], attracting and retaining valuable employees [2, 4, 5, 7], increasing corporate survival prospects during economic turmoil [5, 8, 9], and facilitating entry into foreign markets [5]. A positive corporate reputation translates into competitive advantages and increased market share [10–13]. Finally, a good reputation improves a firm’s financial performance [14–19] and increases its goodwill and market value [1, 16, 17, 20–23]. Consequently, there are considerable studies on corporate reputation presenting various problems from the perspective of reputation management and marketing [6, 17, 24–33]. There are also various models based on the game theory approach that explain the reputation’s role in decision-making problems. However, they tackle simplified structures and often lack the context of decision-making problems. Regarding investment, these models focus rather on games in public goods’ investment [34, 35].

However, a clear gap exists in the literature regarding the interplay between corporate reputation and the behaviour of stock market investors. It is extensively claimed that corporate reputation influences interactions between an organisation and its stockholders and, subsequently, corporate performance [12], but no established framework exists that would enable researchers to study the impact of corporate reputation on the individual behaviours of stock market investors. Filling this gap seems to be essential as corporate reputation influences not only the demand for shares but also stock prices and, along with them, a corporation’s market valuation.

Furthermore, there is no consensus on whether corporate reputation is best conceived of in terms of an individual’s perception of a firm [36] or in more collective terms related to aggregate stakeholder perceptions [10]. Dowling (2016) named these the ‘individual’ and ‘collective’ concepts of corporate reputation. Applying these concepts to stock market behaviour, the former arises from a single investor’s subjective perception of a firm (based upon historical share prices and other available information), while the latter results from the recognition of corporate reputation by all entities in a stock market.

This twofold approach raises some considerations. First, the fact that collective corporate reputation is an aggregation of all stakeholders’ evaluations of individual reputations induces the conclusion that these concepts are not mutually exclusive. Second, assuming that investors behave rationally, they should make decisions not only on individual corporate reputation but also updating it, i.e. considering the collective corporate reputation. Third, other factors could mitigate or moderate the importance of each concept of corporate reputation in the investment decision-making process. For instance, while gaining experience, investors trust their own knowledge and assessment of a given situation, or become increasingly aware of the importance of observing other entities on the stock exchange.

However, popular measures of corporate reputation do not distinguish between individual and collective concepts. As these seemingly similar but different perceptions influence the market valuation of stock listed companies, it is relevant to better understand the way stock investors perceive and process the information about corporate reputation, and finally make their decisions in the stock market.

Consequently, this study is motivated by the concern that these two perceptions, although related, are not driven by the same factors, and thus each may have a distinct impact on the propensity to invest, i.e. the portion of income that investors are willing to invest. The aim of this study is thus to determine the impact of each of the two corporate reputation perceptions on the propensity to invest in the context of decisions made by stock market investors. Identifying which of the two has a greater bearing on investment decisions will benefit further studies of corporate reputation and its impact on investors’ decisions. This study’s results can help avoid misleading research outcomes based on inappropriate reputation measures.

Our study extends the previous literature in three ways. First, we contributed to the discussion on two perceptions of corporate reputation. Second, we focused on individual investors’ perspective rather than on the overall perception of a firm’s reputation which is the focal point in previous studies. Finally, we applied an economic experiment based on vignette studies to analyse individual investors behaviour. To the best of our knowledge, this is the first experimental study comparing the influence of two perceptions of corporate reputation (individual and collective) on stock market investors’ decisions.

The remainder of this paper is organised as follows: the following section provides a brief literature review of two concepts of corporate reputation, and subsequently on investor decision making regarding corporate reputation. In the subsequent section, the method applied is presented with its conceptual background and experimental design. In the penultimate section, data analysis and results are presented. The final section summarises this study and suggests directions for further studies.

## Literature review

### Two concepts of corporate reputation

Haywood claimed that corporate reputation ‘is the perceptions in the minds of those observing the organisation’ [37]. This perception is built over time and focuses on what the organisation does and ‘how it behaves’ [38]. Other researchers claimed that corporate reputation is rather a concept based on what stakeholders think they know about a firm, so it reflects people’s perceptions [10]. It results from perceptions of a firm’s past actions and financial performance [9, 10, 39]. A corporate reputation represents the ‘net’ affective or emotional reaction–good or bad, weak or strong–of customers, investors, employees, and the general public to the company’s name [1]. Reputation can be seen as an individual concept (related to one’s own perception of a firm), a collective, or social concept [40]. Some researchers limited their understanding of corporate reputation and focused on one of these ideas. Gotsi and Wilson, for instance, stated that corporate reputation is a stakeholder’s overall evaluation of a firm [36]. Helm claimed that it is ‘the individual’s perception of the general estimation in which a firm is held, good or bad’ [41]. In other publications, corporate reputation is regarded as an aggregation, a set, or a multi-factor function of all stakeholder perceptions, including perceptions of suppliers, customers, workers, managers, and shareholders [10, 42, 43]. In this regard, it can be defined as ‘a collective representation of firm’s past actions’ [14]. Similarly, Balmer states that corporate reputation is the ‘enduring perception of an organisation held by an individual, group or network’ [44].

The problem of two concepts of corporate reputation raises the question of appropriate corporate reputation measures. From Dowling, who conducted an analysis of 50 definitions of corporate reputation, the individual corporate reputation should be measured as a person’s evaluation. The collective one, which is ‘a group-based construct’ based on evaluations of ‘like-minded people’, could be measured using for example cluster analysis. Finally, the social concept of reputation should be analysed on the basis based on the theory of the group [40].

To prevent the halo effect investors might focus on the aggregation of reputations in the stock market and intentionally avoid information about the perceptions and valuations of other entities. The existence of other entities’ perceptions or valuations of a corporation, which an investor either cannot observe or intentionally avoids, rises doubts whether the concepts of individual and collective reputation are collectively exhaustive.

Studies of corporate reputation, including studies of its improvement and deterioration and the causes and consequences of these changes, usually focus on one stakeholders’ group (‘like-minded people’). This raises a significant concern regarding commonly applied general corporate reputation measures (e.g. Fortune’s The World’s Most Admired Companies, Reputation Quotient, RepTrak, and Reputation Dividend), because reputation may differ among different stakeholder groups [45, 46]. Furthermore, there are not only different corporate reputations in each stakeholder group but also different corporate reputations perceived by each individual stakeholder [47]. The criteria for assessing a firm’s reputation vary depending on stakeholders’ expectations, and what functions they perform in their relations with that firm [11, 45, 46].

For instance, Korn Ferry’s ranking of the World’s Most Admired Companies, which is appealed to in numerous studies [2, 16, 17, 19, 20, 23, 48–50], is compiled by way of a survey wherein respondents (top executives, directors, and industry analysts) are asked to evaluate firms based upon selected attributes. The respondents are told ‘ratings may be based on your firsthand knowledge of these companies or on anything you may have observed or heard about them’ [51]. Besides the problem of the varying significance of the different attributes for each stakeholders’ group, it cannot be excluded that some respondents made their choices based on their perceptions of collective corporate reputation. Consequently, it cannot be ruled out that respondents have different ways of understanding the concept of reputation. Another example is the Corporate Reputation Quotient where interviewees are asked to answer two questions: ‘Of all the companies you know or are familiar with, which two would you say have the best reputations?’ and ‘Of all the companies you know or are familiar with, which two would you say have the worst reputations?’ Further, in this ranking, it cannot be ruled out that respondents have different understandings of corporate reputation as individual perception or as perception of collective reputation.

Another problem was noticed as early as 1988 when McGuire, Sundgren, and Schneeweis found that the assessment of corporate social responsibility based on rankings was strongly correlated with the firm’s previous financial performance [52]. Others have since claimed that the results of the corporate reputation rankings were biased due to the halo effect [48, 50]. While studying the problem of commonly used corporate reputation measurement methods, Nawrocki and Szwajca [53] found them highly subjective and unadjusted to a given stakeholders’ group. As a result, they introduced a model focusing more accurately on the investor’s perspective. This model was designed to provide an objective measure based on available qualitative and quantitative data rather than surveys.

The rankings’ results are important and useful for general studies of corporate reputation. However, in the case of collective corporate reputation, due to the failure to adjust for specific stakeholders’ group, the occurrence of correlations, the halo effect, and the different possible ways respondents conceive reputation, the resulting rankings should be applied with extreme caution. One solution to this problem could be assigning weights to each analysed attribute depending upon their importance to stock market investors. This, however, would not solve the problem of individual corporate reputation measurement.

The consideration of individual and collective corporate reputation of stock-listed companies regarding specific stakeholder group creates a need for a new measure that would allow further analyses of stakeholders’ decisions.

### Investors’ decision making

Previous theories describing the behaviour of stock market investors focused mainly on financial data and the inflow of new information. Starting from Markowitz’s portfolio selection [54], the capital structure model of Modigliani and Miller [55, 56], capital asset pricing model by Sharpe and Lintner [57, 58], Fama’s Efficient Market Hypothesis [59, 60], etc., the early models were based on assumptions of the rationality of decision-making entities and full access to information. Although models supporting investor decisions have been developed, studies have shown that investors’ behaviour does not always correspond with the solutions of these models [61]. Attempts to explain these discrepancies resulted in the development of behavioural finance which incorporates psychological aspects in financial decision making—starting with the famous prospect theory by Kahneman and Tversky [62], the works of Barber and Odean [63, 64], Barberis [65, 66], De Bond and Thaler [67, 68] and Hirshleifer [69, 70]. Additionally, one of the most popular methods for observing and analysing individual investor perceptions, evaluations, and decisions became an economic experiment whereby subjects’ behaviours were motivated by financial incentives and analysed in a controlled environment. Decision-making experiments aiming to investigate stock market investors’ behaviours confirmed the presence of status quo bias [71], overreaction and underreaction [65, 67], expectation extrapolation [72] and many other psychological biases affecting investors decisions.

Asides from the historical share prices and psychological factors, stock market investors consider the behaviour of other market players most important. Therefore, their decisions can be influenced by analysts’ recommendations [73–76] and the behaviours of institutional and individual investors. The investor’s imitation of the actions of others is known as herding behaviour and extensively studied [77–82]. However, when investors cannot properly judge other investors trading abilities, and follow them basing on inappropriate characteristics (e.g. investor’s charisma), herding is not beneficial [83].

Recently, there has been a tremendous increase in research into the significance of corporate reputation to stock prices and firms’ market value [1, 9, 16, 17, 22, 50, 84–89]. However, the relationship between corporate reputation and corporate market value is indirect and non-straightforward. The factor that mediates this relationship, i.e. the factor directly influenced by corporate reputation, is the behaviour of stock market investors which could be expressed in the propensity to invest. This behaviour influences stock prices and firms’ valuations.

Investors’ primary goal is to obtain a sufficiently high rate of return. Knowing that stock prices are in fact influenced by the behaviours of all the stock market investors, it seems rational for an investor to incorporate the information about the others’ behaviour into the decision-making process. This process resembles the game-theoretical approach where players are aiming to predict others’ behaviours—the concept is known from the Keynesian beauty contest [90] in which relying on one’s own perceptions is a naïve strategy. As a result, regardless of whether an investor considers corporate reputation an important variable in their own decision-making process or not, the investor assumes that reputation influences the decisions of other investors. For instance, a better corporate reputation constitutes a signal of a greater interest in its shares in the near future, and therefore a higher probability of achieving greater returns on equity. This mechanism suggests that investors incorporate socio-cognitive factors in their decision-making processes and focus on their perception of collective corporate reputation rather than on their individual assessment of corporate reputation.

We develop the previous studies on the impact of corporate reputation on the behaviour of stock market investors (based mainly on rankings) by considering the impact of other market players (financial analysts and other investors) on investment decisions in the stock market. We predict that investors, having the possibility to assess both, their individual perception of corporate reputation and the perception of collective corporate reputation, will base their investment decisions on the latter. As previous studies confirmed a positive relationship between corporate reputation and market value [1, 16, 17, 20–23], we assume that there should be a positive relationship between collective corporate reputation and the propensity to invest in this company’s stock. Consequently, we state the following two hypotheses.

*Hypothesis 1 (H1)*: *There is a positive relationship between the perception of collective corporate reputation and the propensity to invest in stock*.
*Hypothesis 2 (H2)*: *There is no relationship between the individual corporate reputation and the propensity to invest in stock*.

One of the methods suitable for studying individual investor behaviour and their reactions to changes in corporate reputation is an economic experiment whereby subjects’ behaviours are motivated by financial incentives and analysed in a controlled environment. However, there are only limited economic experiments in the study of corporate reputation regarding stock investors’ behaviours. For instance, an economic experiment method was utilised to investigate the impact of the presentation of performance information on ‘stakeholders’ attitudes towards firms that seek to enhance their reputation’ [91]. Specifically, the effect of selected information characteristics was investigated, i.e. the message source, information type (numeric vs verbal), and reference point (trend vs competitor comparisons) on stakeholder’s attitudes towards the firm. The full factorial design was built on the manipulation of these three characteristics. However, this study mainly focused on the possibilities of managing corporate reputation but not on investors’ behaviour.

In another economic experiment, the probability of buying shares was analysed based on a full factorial design study [74]. The following factors were manipulated: the value of analyst recommendation (neutral vs. positive), reputation value (negative vs. positive) and reputation domain (ethical vs. financial). Each of these factors was proven to be significant for investors’ behaviour.

A series of three experimental vignette studies [92] on the impact of corporate reputation disclosures on stakeholders’ behavioural intentions showed that, in the case of investors, this relationship is fully mediated by their perceptions of organisational performance and corporate reputation. Additionally, corporate reputation disclosures mitigate uncertainty about future stock prices and thus ‘reduce the risk perception of a future investment’. The application of experimental vignette studies, which are based on situational descriptions, allowed researchers to manipulate the levels of selected variables and observe investors’ behaviour in each experimental group.

Furthermore, as with any other behaviour, making investment decisions by processing company-related information and data on other stakeholders’ behaviours is subject to some specific factors. For instance, former experience in investing and gender can affect the perception of collective corporate reputation and thus the investment decisions. Young and less educated investors with lower incomes and wealth trade more often, with higher-stakes, and tend to have less diversified portfolios. Consequently, they tend to achieve lower trading performance [93]. Considerable studies, based on Bayesian belief learning and reinforcement learning show that trading experience significantly helps investors achieve higher portfolio performance [94–96]. Previous studies suggest that more experienced investors are less affected by behavioural biases [93, 97–99]. However, while gaining experience under naive reinforcement learning, investors can overestimate the importance of their personal experience [100]. Additionally, the experience differs for individual and institutional investors. Individual investors are considered to be more uninformed and unskilled, and they make various mistakes [95, 96]. Consequently, their learning behaviours are even more interesting for analysis.

Trust is also an important factor regarding investment decisions. Previous experimental evidence suggests that women are less trusting when making investment decisions [101]. Questions also arise about whether trust may influence perceptions of corporate reputation in the market. An individual’s propensity to invest may also be influenced by their attitudes towards risk. Previous studies have shown that a higher willingness to take risks results in greater interest in stocks and the possession of more assets and riskier assets [102].

Decisions made by investors, especially individual investors, can be further influenced by their current economic situation. However, when conducting surveys, questions about participant’s incomes make them feel rather uncomfortable [103]. Besides, the current income does not always reflect the real economic situation of the respondents, especially regarding students or older people [104]. Consequently, some authors have argued that it is not only the absolute income that matters but also the relative value [105, 106]. This approach to measuring the income variable is applied in numerous decision-making studies in not only in economics, but also in sociology [107], psychology [108] and medicine or health sciences [109]. The analysed variables, consistent with this approach (although based on various measures), are subjective economic position [108, 109], perceived economic situation or subjective perception of the economic situation [109], subjective perception of their socio-economic situation [108], and subjective perception of income [107].

Based on the above considerations, when attempting to evaluate collective corporate reputation, it seems reasonable to control for previous experience in investing, gender, and trust in the model. When attempting to model changes to the individual propensity to invest, it would be beneficial to control not only for gender and investment experience but also for risk attitudes, an investor’s subjective economic situation, and the value of the investor’s current holdings in the analysed company.

There are various studies on investor decisions and the factors on which they depend: financial, psychological, and sociological. However, the impact of corporate reputation, both individual and collective, and their interactions with other factors still require further research.

## Method

This study project’s approval was waived by the Ethics of Scientific Research Committee at the University of Gdansk, Poland. The participants were guaranteed anonymity. Their participation in the study was voluntary and they were free to withdraw at any time.

### Conceptual background

In this study, we focused on individual investor’s financial decision-making processes which can be affected by different concepts of corporate reputation. Consequently, we are consistent with the two definitions claiming that corporate reputation is a stakeholder’s overall evaluation of a firm [36], and that it reflects people’s perceptions [10]. These two definitions respectively, constitute individual corporate reputation and the perception of collective corporate reputation in our study. This is especially worth emphasizing that we do not analyse the collective perception of corporate reputation (which could be measured for example by cluster analysis [40]) but the investor’s perception of collective reputation, which influences their propensity to invest. This term plays a focal role in our study. We claim that individual investors’ perceptions of collective reputations influence their investment decisions. Furthermore, the context of investors’ behaviour is expressed in the definition proposed by Petkova [110] which states that reputation is stakeholders’ perceptions about a firm’s ability to deliver value.

**Fig 1:**
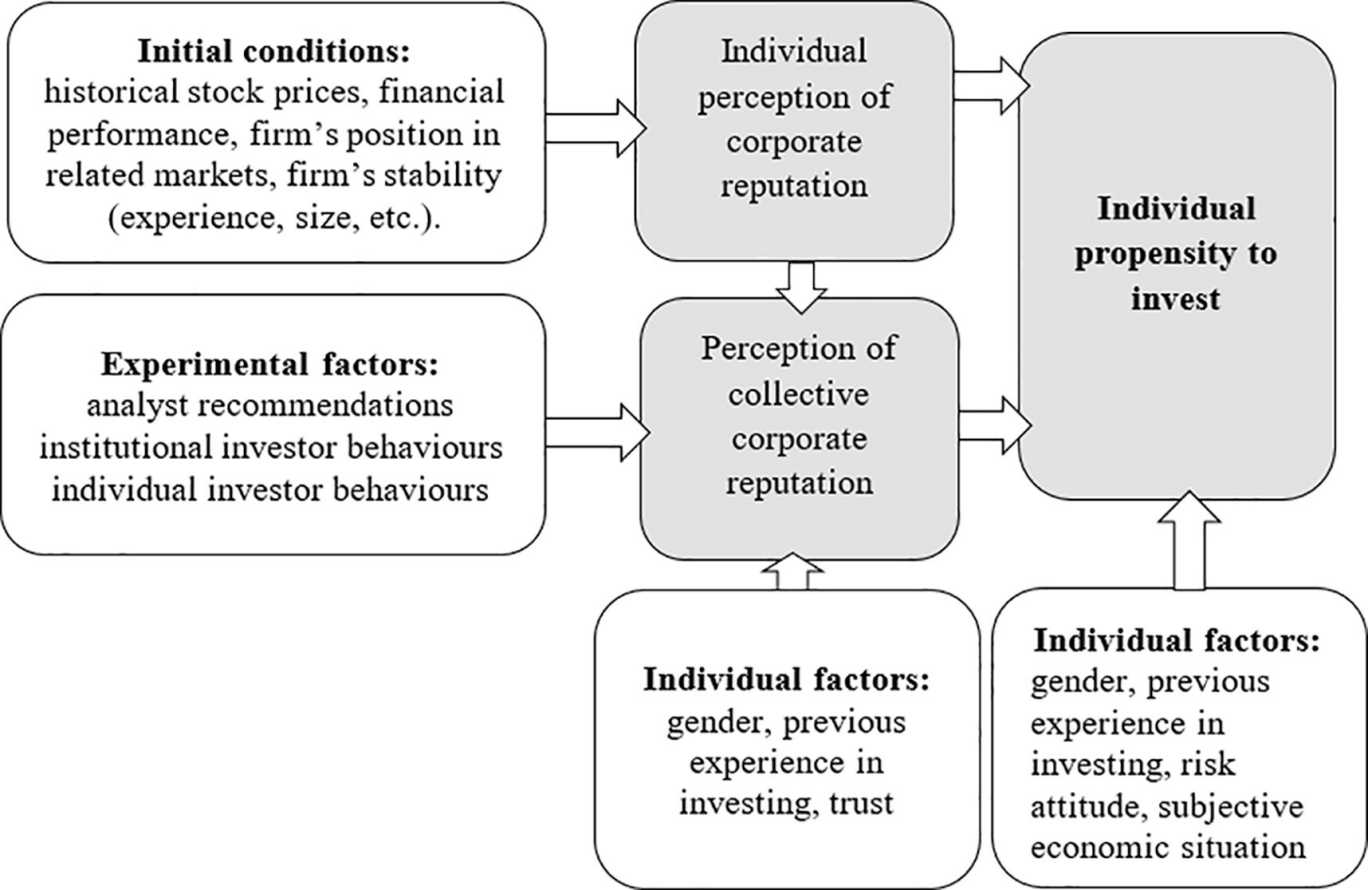
Experimental design: A conceptual model.

In this study design, the concept of individual corporate reputation (ICR) is based mainly on an individual’s perception of a firm’s past performance and actions (Fig 1). It was induced by the experimental setting (initial conditions), in which a selected firm’s characteristics and historical stock prices were presented. The participants were informed that they would analyse a large company operating in the service industry for 16 years. Financial analysis shows that the values of the company’s financial ratios (liquidity, profitability, debt and turnover) are within industry standards. The sector is very stable. The company has been listed on the stock exchange for nine years. Participants could see the chart with stock prices that were slightly fluctuating around an increasing trend and during the last 10 days the prices increased by 11%. All participants received identical information.

The concept of perception of collective corporate reputation results from each investor determining a corporate reputation among members of a social group from the behaviours of other entities. In this study design, participants determine the perception of collective corporate reputation by observing analyst recommendations and institutional and individual investors’ decisions. We distinguished institutional investors, as it is claimed that ‘central and more prestigious active institutional investors may serve as a certification provider’ for a company, confirming the possibility of increasing its value in the future [111].

Furthermore, consistent with the definitions, we assumed that each investor’s perception of collective corporate reputation was also influenced by their perception of corporate reputation–the individual corporate reputation (ICR; Fig 1). These ideas are expressed, for instance, in the Wartick’s definition from which corporate reputation is ‘the aggregation of a single stakeholder’s perceptions of how well organisational responses are meeting the demands and expectations of many organisational stakeholders’ [112]. We decided to induce the perception of collective corporate reputation by applying a factorial approach based on situational descriptions (vignettes) [92, 113, 114]. Specifically, we manipulated the following three factors intending to influence the perception of collective corporate reputation: analyst recommendations, institutional investor behaviours, and individual investor behaviours.

The assessment of ICR and perceived collective corporate reputation and the following investment decisions made by participants in the experiment enabled us to observe the relationship between ICR and propensity to invest in stock, as well as the relationship between the perception of collective corporate reputation and propensity to invest in stock.

Additional questionnaires in this study enabled us to control for previous experience in investing, gender, and trust in the model that checks the manipulation–the model of perception of collective corporate reputation. Furthermore, it enabled us to control for gender and investment experience, risk attitudes, an investor’s subjective economic situation, and the value of the investor’s current holdings in the analysed company in the main model of individual propensity to invest.

### Experimental design

The experiments’ main advantage over other research methods is that the research is conducted in a controlled environment in which the researcher can set up institutions and manipulate selected variables. This enables the researcher to analyse the influence of selected variables on investor’s decisions [115]. An analysis of the same variables in the real world could be disrupted by other factors beyond the researcher’s control and observation. An experimental design based on vignettes establishes a more realistic scenario [113], and a context that allows the incentives of participants to align more with the incentives of real investors than experiments based on the game theory approach.

Our incentivised vignette study was designed to reflect stock market conditions. Subjects could observe a firm in an artificial stock market, assess its reputation, and invest artificial money. To achieve reliable data, we conducted an online experiment as online respondents feel more comfortable and are opportune to make decisions in a convenient, natural location and time (albeit within a specified period), thus making decisions that closely resemble their likely decisions under actual conditions. Due to the advantages of online experiments outweigh their disadvantages, online experiments are becoming increasingly popular [116, 117].

When starting the game, participants were presented with the ‘introduction for participants’ explaining the main idea of the study, time duration, privacy conditions, etc. Subsequently, they were asked whether they voluntarily consent to participate. Detailed rules for the experiment were presented to those who agreed to participate in the study. In order to obtain the highest quality data, we asked participants to take part in a quiz designed to assess the extent to which they understood the rules of the experiment. The results allowed us to exclude entries from participants who did not correctly understand the rules.

**Fig 2:**
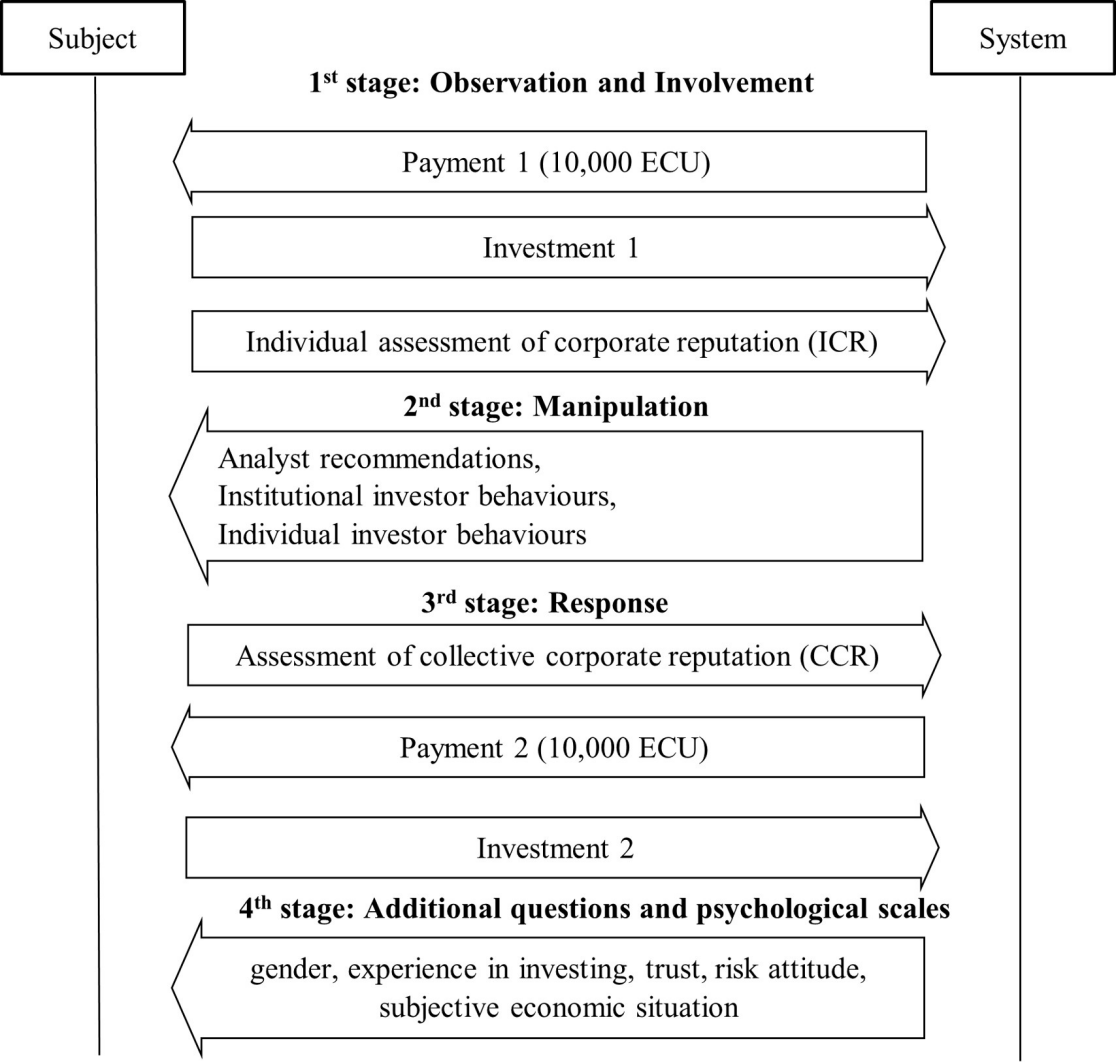
Experimental design: Timeline and information flow between the subject and the system.

The experiment occurred in four stages. At the beginning of the first stage–Observation and Involvement–subjects were endowed with 10,000 ECU (experimental currency units) that they were informed that they would later have the opportunity to invest (Fig 2). Subsequently, subjects observed an experimental environment where information about a company was displayed, including historical stock prices, financial performance, position in related markets, and stability (i.e. experience, size, etc.). All participants were presented with the same company with identical characteristics, which enabled us to maintain the formal equivalence among the experimental groups and observe the unbiased results of further manipulation [92, 114]. This study was based on a fictitious company to avoid biased evaluations based on prior attitudes towards a real-life company [8, 92].

The information about the firm was intended to influence and constitute each subject’s individual corporate reputation. At the end of this stage, each investor could invest money. The ratio of investment to income (10,000 ECU) reflects an investor’s propensity to invest. As all participants received identical information, the variable’s volatility can be interpreted as a natural difference in the propensity to invest among individual participants. Furthermore, each participant was asked to rate individual corporate reputation on a Likert scale (ICR∈〈0,10〉) by answering the question ‘How do you rate the reputation of this company?’

In the second stage–manipulation–we manipulated the following three factors intending to influence the perception of collective corporate reputation: (1) analyst recommendations (‘buy’ or ‘sell’), (2) institutional investor behaviours (where institutional investors are reported to be either more or less interested in a company’s shares), and (3) individual investor behaviours (where individual investors are reported to be either more or less interested in a company’s shares). At the start of the experiment, each participant was randomly assigned to one of the eight experimental condition groups—resulting from three experimental factors, each at two levels (2^3^). In the manipulation stage, each participant was presented with the information specified for that group, i.e. they were informed that financial market analysts were recommending to either buy or sell shares of an analysed company, that institutional investors were becoming either more or less interested in this company, and that individual investors were becoming either more or less interested in this stock.

In the third stage–response–subjects utilised their observations of other entities behaviours (analysts, institutional, and individual investors) to rate the collective corporate reputation on a Likert scale (CCR∈〈0,10〉). Particularly, they were asked the following question: ‘In your opinion, how is the corporation’s reputation rated in the stock market?’ The answers to this question constituted the perception of collective corporate reputation (CCR). Each investor then received an additional 10,000 ECU. At the end of this stage, each investor had to decide whether to invest money in shares of the analysed firm. On the basis of investment decisions, we calculated their new ratios of individual propensity to invest.

In the fourth stage, subjects answered additional questions concerning their demographical and psychological characteristics. The information provided enabled us to control for individual factors in the model, such as gender, previous experience in investing, trust, risk attitude, and participant’s subjective economic situation. There are various measures of trust, and they are divided into direct and indirect measures. Direct measures are based on self-reporting, and subjects can be asked about the extent to which they trust people. For example, Naef and Schupp proposed an indicator based on three statements and one question, all of which were rated on a four-point scale [118]. Indirect measures of trust are based on observations of people’s reactions, behaviours, and decisions [119]. In this study, we applied direct measures because of their simplicity and comprehensibility to the respondents. Additionally, its construction corresponds with the construction of corporate reputation perceptions applied. As a measure of trust, we used the first sentence from Naef and Schupp’s test [118], which considers general trust: ‘Generally, most people can be trusted’. Subjects were asked to indicate the degree to which they agreed with this sentence on a 4-point scale (ranging from ‘disagree strongly’ to ‘agree strongly’).

There are many risk-measurement methods. Their complexity enabled researchers to distinguish three main groups: behavioural measures of risk, measures of risk attitude, and measures of personality traits related to risk. The first group comprises measures based on real and hypothetical choices, including methods such as BART that are based on visualisation [120] and methods that utilise lotteries [121]. The second group comprises measures based on self-report questionnaires, including descriptions and questions concerning risky situations such as DOSPERT [122]. The third group comprises measures based on self-reports of personality traits related to risk attitudes, such as the need for arousal [123] and resistance to change [124].

One of the subjective risk-assessment methods, the risk question from the German Socio-Economic Panel Study (SOEP) [125, 126], has been applied in studies concerning investment decisions [102, 127]. This simple risk-elicitation method was found to be ‘a good predictor of actual risk-taking behaviour’, one that has considerable predictive power and overperforms incentivised lottery measures [125]. These are the main reasons why we applied this direct measure of risk attitude. To assess individual risk attitudes, each subject was presented with the following question: ‘How do you see yourself: Are you generally a person who is fully prepared to take risk, or do you try to avoid taking risk?’ Subjects were subsequently tasked with classifying themselves using an 11-point scale from 0 to 10, where 0 indicates ‘unwilling to take risks’ and 10 indicates ‘fully prepared to take risks’. The method’s main advantages are its simplicity, clarity for respondents, and reliability, as confirmed by previous studies [125, 126].

Regarding the economic situation, because people with the same income may have different expenditures and different social environments, we maintained that the absolute value of income is not an appropriate measure of the economic situation. Consequently, in this study, we decided to ask subjects to rate their own economic situation on a 5-point scale (ranging from 0 - ‘very bad’ to 4 - ‘very good’) in response to the following question: ‘How would you rate your current economic situation?’ The answer to this question was used as a measure of the subjective economic situation of an investor.

At the end of the game, participants learned their monetary outcome, and ‘winners’ received a unique code entitling them to receive the voucher. We applied an incentivised study in which participants could earn a voucher for a popular online shop (worth the equivalent of 12 EUR), only if they earned in the experiment more than the average amount earned by subjects (N = 60) in the pilot study. This voucher was chosen earlier in a survey.

## Data analysis and results

Based on interviews with participants in the preliminary study, we adjusted the comprehensibility of our study design (vignettes, rules of the experiment, etc.) for the participants. Participants in the main study (*N*_0_ = 660), who were recruited from multiple universities in Poland, were either students in their final year of master’s studies in economics and finance or students in the second semester of their master’s studies in the same fields, who had both passed courses related to stock markets and engaged in laboratory trading based on actual data. To obtain the highest quality data, we excluded entries of the participants who had difficulties passing the quiz on the rules of the experiment. We also excluded records of the participants whose task-execution time was too short or too long compared to the pilot study, as we assumed some participants could be careless in their approach to the experiment. In the final sample (*N* = 528), there were 34% male subjects (Table 1). In this study, we had some considerations regarding including real experience in trading at a stock exchange, as our participants were students. However, included this predictor as 6% of the survey participants had some experience in stock market investment, ranging from one month to five years. Furthermore, 45% of participants were part-time students (many of them with work experience), and 23% of all students were 25 years or older. Mean age was 24.2 (Me = 23; max. = 55). Finally, the number of participants assigned to each of the eight experimental conditions varied from 61 to 70.

**Table 1:** Summary statistics and pairwise correlations matrix for the CCR model.

Variable	M	SD	Min	Max	gender	exp	trust	ICR
experience	0.98	6.01	0	60	0.16***	.		
trust	2.06	0.70	1	4			.	
ICR	7.80	1.45	2	10	-0.08*			.
CCR	6.61	1.93	0	10				0.42***

Pearson correlations between variables *** p < .001, ** p < .01

* p < .05

**Fig 3:**
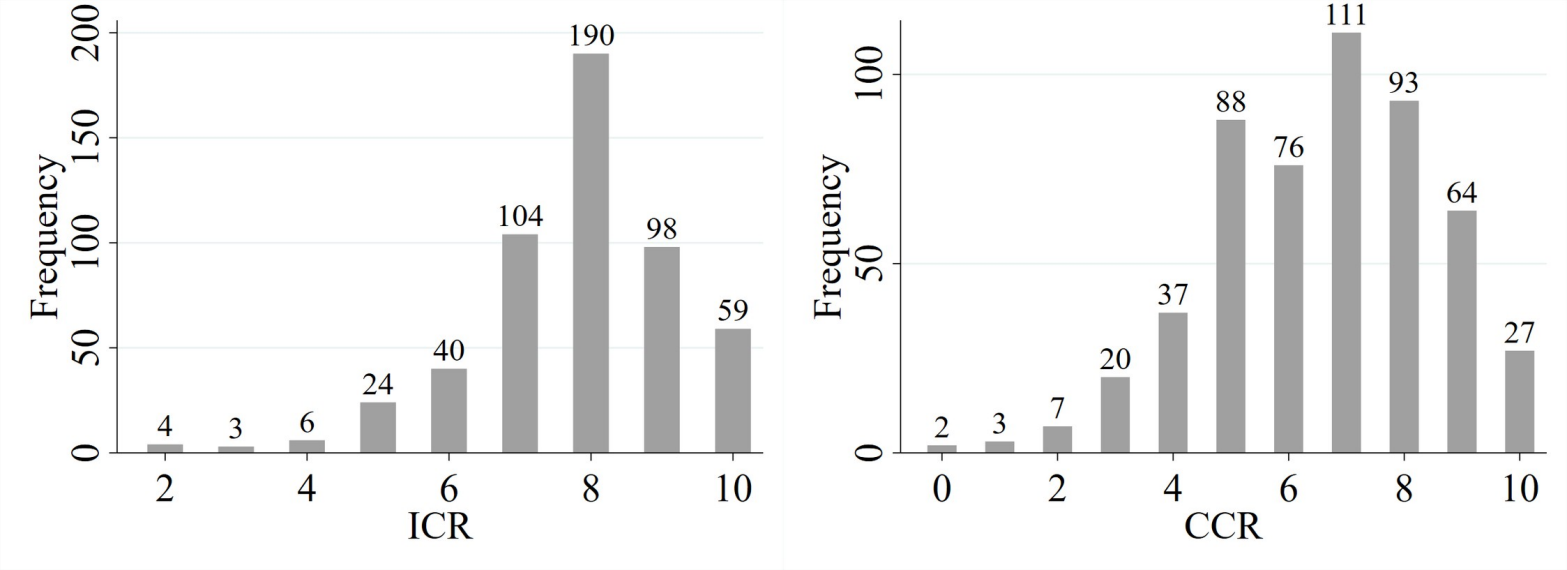
Individual Corporate Reputation (ICR) and perception of Collective Corporate Reputation (CCR).

Assessments of individual corporate reputation and the collective corporate reputation were relatively high (ICR: M = 7.80, Me = 8; CCR: M = 6.61, Me = 7; Table 1) which resulted from the company’s positive image built by initial conditions (experimental settings). However, in the case of CCR, higher dispersion was observed (Fig 3) as investors could observe diversified information on the behaviours of other stakeholders (ICR: SD = 1.45; CCR: SD = 1.93; Table 1).

Verifying the outcomes of the manipulation used, we regressed CCR on analyst recommendations, institutional investor behaviours, and individual investor behaviours. Additionally, we controlled for gender, experience, and trust. We also included ICR in the model. The best-fitting regression model was found to be ordered logistic regression—a model often applied in behavioural studies.

All three social factors, i.e. analyst recommendations (Coef. = 0.665, p = 0.000), institutional investor behaviours (Coef. = 0.763, p = 0.000), and individual investor behaviours (Coef. = 0.811, p = 0.000; Table 2), were found to be statistically significant in the final model.

**Table 2:** The perception of collective corporate reputation.

CCR	MODEL 1			MODEL 2		
	Number of obs = 528			Number of obs = 528		
	LR chi2(5) = 179.08			LR chi2(5) = 171.67		
	Prob > chi2 = 0.0000			Prob > chi2 = 0.0000		
	Pseudo R2 = 0.0832			Pseudo R2 = 0.0798		
	Coef.	SE	P	Coef.	SE.	p
analyst recommendations	0.643	0.157	0.000	0.665	0.157	0.000
institutional investor behaviours	0.767	0.157	0.000	0.763	0.157	0.000
individual investor behaviours	0.839	0.159	0.000	0.811	0.158	0.000
ICR	0.674	0.060	0.000	0.673	0.060	0.000
gender	0.329	0.166	0.047	-	-	-
experience	0.017	0.013	0.198	-	-	-
trust	0.104	0.110	0.348	-	-	-

Regarding control variables, in the initial model (Table 2, MODEL 1), experience and gender were included. However, they were correlated with each other, and gender correlated with ICR (Table 1). Even excluding gender and ICR, experience was not statistically significant. In the final model, there were no effects of trust, gender, or previous experience with investing on the perception of collective corporate reputation (CCR).

Although gender was statistically significant in the primary model (Table 2, MODEL 1) and previous analysis showed a weak (p < 0.5) negative correlation between gender and ICR (Table 1), there were no significant interactions of gender with other variables found in the final model. ICR proved relevant for CCR (Coef. = 0.673, p = 0.000). The results suggest that when individual investors assess the collective corporate reputation, they consider other stakeholders’ perceptions of corporate reputation, but their assessments are also anchored in their own views of that corporation, i.e. in ICR.

Then we moved to the main model of study–the propensity to invest model. Analysing the pairwise correlations matrix (Table 3), we found a positive relationship between the perception of collective corporate reputation and propensity to invest in stock and no relationship between the perception of individual corporate reputation and propensity to invest in stock. These results confirmed our hypotheses (H1 and H2).

**Table 3:** Pairwise correlations matrix for the model of propensity to invest.

Variable	propensity to invest	CCR	ICR	invest1	gender	experience	risk
CCR	0.80*	.					
ICR		0.42***	.				
invest1	-0.36***	0.16***	0.22***	.			
gender			-0.08*	0.11**	.		
experience					0.16***	.	
risk				0.09**	0.09**		
SES			0.08*	-0.07*			0.08*

Pearson correlations between variables *** p < .001

** p < .01

* p < .05

Subsequently, we applied ordered logistic regression (Table 4), the best fitting regression model in this case, in which our dependent value was at three levels: increase in propensity to invest, no change in the propensity to invest, and decrease in the propensity to invest. We included ICR in the model to check if it became significant due to interactions with other predictors. Furthermore, we controlled for the value of the first investment (invest1), gender, experience, risk attitude, and participant’s subjective economic situation (SES).

**Table 4:** Change in the propensity to invest.

Change in the propensity to invest	MODEL 1			MODEL 2		
	Number of obs = 528			Number of obs = 528		
	LR chi2(5) = 66.43			LR chi2(5) = 60.08		
	Prob > chi2 = 0.0000			Prob > chi2 = 0.0000		
	Pseudo R2 = 0.0702			Pseudo R2 = 0.0635		
	Coef.	SE	p	Coef.	SE.	p
CCR	0.182	0.055	0.001	0.179	0.050	0.000
ICR	0.005	0.073	0.947	-	-	-
invest1	-0.224	0.031	0.000	-0.210	0.030	0.000
gender	0.232	0.196	0.235	-	-	-
experience	-0.003	0.015	0.862	-	-	-
risk attitude	0.062	0.037	0.097	-	-	-
SES	-0.212	0.132	0.109	-	-	-

As no statistically significant interactions were found between predictors in the model, we focused on the MODEL 1 –without interactions (Table 4). The relatively low values of R-squared coefficients found in all our logistic regression models–that is 8.32% and 8.20% in models explaining CCR (Table 2) and 7.02% and 6.35% in the model explaining propensity to invest (Table 4)–are also frequently observed in other studies based on primary data [128]. Such low values are therefore common in behavioural studies and generally in microeconometrics [129, 130], while models estimated for time series and aggregates (used in macroeconomics) are characterised by a higher R-squared [130]. Furthermore, the study results reported in Stata are based on McFadden’s R-squared, which results in values smaller than the regular R-squared (Likelihood Ratio Index; LRI).

In the final model (MODEL 2, Table 4), CCR was found to be a significant predictor of changes in the propensity to invest (Coef. = 0.179, p = 0.000), whereas in the case of ICR no significant effect was found. The other statistically significant predictor was the amount previously invested (Coef. = -0.210, p = 0.000). Thus, our findings support both hypotheses in this study. Subjects who had already invested (invest1) relatively more money in stock than others were less likely to increase their propensity to invest (Coef. = -0.224, p = 0.000). Furthermore, regarding the propensity to invest, we found no statistically significant effects of gender, previous experience in investing, risk attitude, or subjective perception of one’s economic situation.

## Summary and conclusions

Corporate reputation is undoubtedly decisive for stakeholders’ decisions. In this study, we determined the importance of two corporate reputation concepts (individual and collective) for stock market investors in an incentivised economic experiment based on a vignette study. Our results support the first hypothesis, which states that there is a positive relationship between the perception of collective corporate reputation and propensity to invest in stock. They also support the second hypothesis, as we discovered no significant relationship between the individual corporate reputation and propensity to invest in stock.

The experimental results suggest that the perception of corporate reputation among real investors is shaped not only by the observed behaviours of other market participants (analysts and institutional or individual investors), but it is also anchored in investors’ own initial valuation of corporate reputation. Thus, advocating that investors update their perceptions, or even change them, under the influence of the observed behaviours of other market participants. This result is consistent with Dowling’s (2016) research results, from which most researchers relate their definitions of corporate reputation to the collective approach (38 out of the 50 analysed definitions). Consequently, the perception of collective corporate reputation can be recognised as an updated individual’s initial perception of a firm’s reputation. This line of reasoning requires further studies of the mechanism of updating these perceptions. To sum up all these perceptions, one should first find an objective measure of each individual’s perceptions and decisions. An economic experiment can supply such a measure by enabling the analysis of individual investor behaviours. Moreover, a controlled economic experiment enables the observation of how corporate reputation is built and how it can affect investor decisions.

Interestingly, although corporate reputation is based on the observation of others’ behaviours, general trust in people had no significant impact on perceptions of corporate reputation in the market (CCR). Application of alternative trust measures in further studies can allow researchers to verify the meaning of this factor for the relationship between corporate reputation and the propensity to invest.

This study revealed that the more money subjects had already invested in stock, the less probable it was that they would increase their propensity to invest in the experiment (even if they received additional money). However, the previous experience in investing was not a significant predictor of investment decisions, which may induce the conclusion, that people’s investing behaviour is strongly rooted in their psychological traits and their current perception of a given situation.

The main limitation of this study is that it proved impossible to recruit a sufficient number of actual investors to participate in the experiment. A considerable number of participants were needed for each of the experiment’s eight treatments, which would enable us to conduct statistical analysis, so instead we utilised students as participants. This limitation is related to the participants’ experience of investing, especially when reinforcement learning theory is considered [95, 96]. Furthermore, there are differences in the updating of expectations by young and older investors [94], which could also distort the study’s results based on relatively young participants. Thus, the study’s results should be treated with caution. However, we made a significant effort to ensure that the students in question had extensive knowledge of the functioning of stock markets. Furthermore, the question in this case is not about the level of knowledge on investing but about how corporate reputation affects behaviour, and we believe that (as proved in the model) the effect of corporate reputation on investor decisions does not depend significantly on investor experience.

The mediating or moderating role of experience could be relevant to the relationship between each of the two perceptions of corporate reputation and the propensity to invest. It constitutes a field of further research. Future work should include experiments with real investors and analyses of real investors decisions when perceptions of corporate reputation change.

Conclusively, the existence of considerable definitions of corporate reputation induces situations in which researchers studying seemingly the same problem may use different proxies for corporate reputation and arrive at different conclusions. Regarding the impact of corporate reputation on stock investor decisions, previous studies have been based mainly on general measures of corporate reputation and its importance to shareholders. As a result, there is no worked-out scheme that would enable researchers to analyse the perception of corporate reputation by stock investors particularly.

This study showed that investors do not solely trust their own perception of corporate reputation. Their investment decisions are also based on updated beliefs–what they think other people think about corporate reputation. Their decision-making processes incorporate socio-cognitive factors. Regardless of whether investors consider a firm’s reputation an important determinant of their own investment decisions, they assume that reputation influences the decisions of other investors and then stock prices. Consequently, in surveys regarding corporate reputation, participants should not be asked about their opinion of the company, but rather about what investors think of the company’s reputation and perceptions of corporate reputation in the stock market. That is, they should be asked about collective corporate reputation.
